# Evaluating Risk in Kidney Living Donors

**DOI:** 10.3389/ti.2025.14024

**Published:** 2025-02-26

**Authors:** Fernanda Ortiz, Lorna Marson, Rachel Thomas, Andreas Kousios, Elvana Rista, Carmen Lefaucheur, Sanem Cimen, David Cucchiari, Gianluigi Zaza, Lucrezia Furian, Baris Akin

**Affiliations:** ^1^ Abdominal Unit, Nephrology, Helsinki University Hospital, Helsinki, Finland; ^2^ Edinburgh Transplant Centre, Royal Infirmary of Edinburgh, Edinburgh, United Kingdom; ^3^ School of Medicine, European University of Cyprus, Engomi, Cyprus; ^4^ Department of Nephrology, Dialysis, and Kidney Transplantation, Hygeia International Hospital, Tirana, Albania; ^5^ Saint Louis Hospital, Assistance Publique- Hopiteux de Paris and Université Paris Cité, Paris, France; ^6^ Department of General Surgery, Sağlık Bilimleri Üniversitesi, Ankara, Türkiye; ^7^ Renal Transplant Unit, Department of Nephrology and Kidney Transplantation, Hospital Clínic, Barcelona, Spain; ^8^ Nephrology, Dialysis and Renal Transplant Unit, Department of Pharmacy, Health and Nutritional Sciences, University of Calabria Rende, Calabria, Italy; ^9^ Kidney and Pancreas Transplantation Unit, Department of Surgical, Oncological and Gastroenterological Sciences, University of Padova, Padova, Italy; ^10^ Department of General Surgery, Demiroglu Bilim University and Group Florence Nightingale Hospital, Istanbul, Türkiye

**Keywords:** end-stage kidney disease, cardiovascular disease, kidney transplant, living donor, hypertension

## Abstract

Kidney donation is a safe procedure for carefully screened donors. The growing shortage of organs and improved survival rates among recipients of living donor transplants have broadened the criteria for acceptable living donors, including older individuals and those with pre-existing health conditions. Consequently, ensuring both the short- and long-term safety of living donors is of paramount importance. The primary objectives are to prevent the need for kidney replacement therapy, major cardiovascular events, or premature death. Lifelong monitoring of living donors is essential to facilitate early treatment for preventable illnesses. To this end, annual follow-up is generally recommended, which should minimally include an assessment of blood pressure, body mass index, kidney function, albuminuria, lifestyle factors, and general wellbeing. However, the management of these risk factors and treatment targets in this population remain inadequately defined. Recommendations for genetic counseling in cases of living-related donation also remain inconsistent. The aim of this mini-review is to address the challenges in evaluating the evidence on the long-term consequences of kidney donation, particularly concerning the risk of developing end-stage kidney disease, cardiovascular mortality, gestational complications, and hypertension. This article aligns with the ESOT call for action to promote living kidney donation and EKITA’s mission.

## Introduction

Globally the number of individuals with end-stage kidney disease (ESKD) has increased with a growing number of patients waiting for a kidney transplant. Even in countries with the highest transplant activity, around 10 patients die every day waiting for a kidney [1]. One way to improve patients’ prognoses is to increase the number of living donor (LD) transplants. Compared to transplants from deceased donors, LD kidney transplants significantly improve recipients’ long-term physical, biochemical, and psychological outcomes [2]. These benefits are maintained even in older LD grafts as they improve graft and patient survival compared to both standard criteria donor and extended criteria donor kidneys or remaining on dialysis [3].

A nephrectomy inevitably results in some health detriment to the voluntary donor, at least in the short term. Potential kidney donors are thoroughly informed about the associated risks. A multidisciplinary team assesses their suitability for the procedure following an extensive health examination [4], and final approval from local authorities. Studies indicate that 86%–98% of kidney donors would choose to donate again [2, 5]. The health risks to the donor are minimal compared to the significant benefits to the recipient.

**FIGURE 1:**
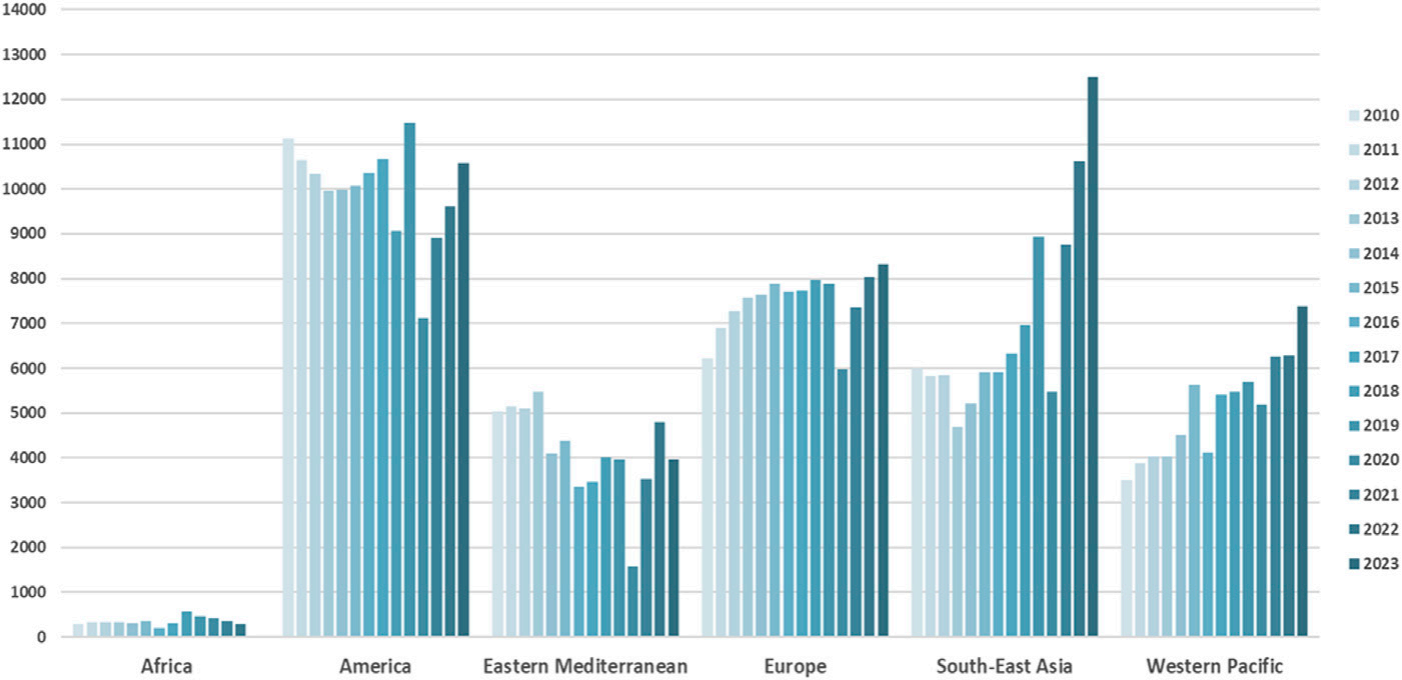
Trends in living kidney donation worldwide. Source: www.transplant-observatory.org.

LD rates increased by 7.8% in 2023 compared to 2022, although with a marked variation in global rates. LD activity has varied across Europe and within countries in the past decade [1] (Figure 1). The variance in the activity is evident not only between countries but also between institutions within the same country. This can be explained by different legal frameworks, socioeconomic, cultural, and religious backgrounds of potential donors, and concerns about the donor candidate’s age and comorbidities influencing acceptance criteria. As the number of global LD kidney transplants increases, it is beholden on the transplant community to continually reassess risk to donors, particularly as the criteria for eligibility for living donation expands; with an increasing number of older donors, or acceptance of co-morbidities that would not have been exclusions 10 years ago. This is the purpose of this literature review.

### Challenges in Interpreting Literature About Living Kidney Donors’ Long-Term Consequences

Live donors represent a unique subset of the population before and after donation. After nephrectomy, kidney donors should be healthy individuals albeit with only one functioning kidney. Defining a comparable population is challenging; thus, risk assessments in the literature should be approached critically. Nowadays, surgical complication rates are low thanks to development of surgical techniques. Recovery from nephrectomy is typically swift, with discharge occurring approximately 2-3 days post-procedure and a return to normal life within 3–6 weeks. However, it is equally important to evaluate the long-term health impacts of kidney donation, particularly the donor’s risk of progressing to end-stage kidney disease (ESKD) or increased cardiovascular risk due to reduced kidney function. It is also necessary to assess whether kidney donation causes psychological harm or reduces quality of life. When compared to the general population, kidney donors tend to have better survival and health outcomes; likely because donors are well-screened healthy individuals, whereas the general population includes individuals with various pathologies [6]. Conversely, compared to individuals who could have donated a kidney but did not, the risks for kidney donors seem to be higher, although there is still controversy [7]. Significant challenges in comparisons arise from varying acceptance criteria for kidney donation, incomplete follow-up data, insufficient registry data, and inadequate consideration of genetic predisposition, smoking, biometric, or socioeconomic parameters in the comparison group. A particular problem is data scarcity on long-term donor outcomes (i.e., studies of more than 15–20 years of follow-up), which makes risk assessment for younger potential donors difficult.

#### Risk of Progressing to End-Stage Kidney Disease

After nephrectomy, the number of nephrons is reduced by half. Serum creatinine rises, and the estimated glomerular filtration rate (eGFR) immediately drops after around 50% reduction in kidney mass [8]. Unlike unilateral nephrectomy in individuals with comorbidities, the LD remaining kidney has adequate kidney functional reserve capacity, which enables compensatory, adaptive hyperfiltration, typically increasing its function in the months post donation. About a year after nephrectomy, kidney function stabilizes at approximately 60%–65% of the initial pre-operative function. Similarly, eGFR decreases after surgery regardless of baseline levels, age, and gender, remaining stable long-term, as in healthy non-donors [9]. The annual eGFR decline was 0.35 mL/min/1.75 m^2^ in donors compared to 0.85 mL/min/1.75 m^2^ in healthy controls in a retrospective matched cohort study of 604 Canadian donors from 2002 to 2016, a difference attributed to donor glomerular hyperfiltration in the first five years post donation. A Dutch registry-based analysis confirmed these findings, although it was noted that in approximately 13% of donors, the expected increase in eGFR post-nephrectomy was not observed [10]. These findings suggest that in some individuals the kidney functional reserve capacity is decreased, perhaps due to factors such as low nephron mass and low birth weight, preventing enhanced function in the remaining kidney, The risk of progressing to ESKD after kidney donation is minimal, occurring in less than 1:200 donors (0.5%) [11]. This risk is significantly lower than in the general (unscreened) population. Muzaale reported on the long-term follow-up of 96,217 kidney donors in the United States, comparing the outcomes to a control population of 20,024 participants from the NHANES III study [12]. Ninety-nine of 96,217 donors (0.1%) developed ESKD on average 8.6 years after donation compared to 36 of 96,217 (0.04%) matched healthy non-donors. Based on this, the estimated risk of ESKD 15 years after donation was 30.8 per 10,000 donors and 3.9 per 10,000 controls. On further analysis of the same registry data, 10 per 10,000 donors developed ESKD within 10 years post donation, primarily due to glomerulonephritis. Twenty-five years post-donation, 85 out of 10,000 donors had developed ESKD, mainly due to diabetes and hypertension [13].

Mjøen reported on the long-term kidney function of 1,901 Norwegian donors, comparing transplant registry data to 32,621 individuals who could have but did not donate a kidney [14]. The average follow-up was 15.1 years for donors and 24.9 years for non-donors. The risk of ESKD was 11.38 times higher in kidney donors. Notably, this elevated risk is based on only nine donors requiring kidney replacement therapy 18.7 years after donation, with seven of these recipients being first-degree relatives of the donors. Similarly, in the U.S. study [12], the authors found that 67% of donors who developed ESKD were biologically related to their recipients. In contrast, most controls had no family history of kidney disease.

Assessing the genetic predisposition to kidney disease is advisable in selected cases when donor and recipient are first-degree relatives [13]. When the recipient’s kidney disease is known, specific cases in which genetic testing might be considered include Alport’s, aHUS, hereditary focal and segmental glomerulosclerosis, Fabry’s, and autosomal dominant polycystic kidney disease. However, this approach remains a matter of debate and there is wide variation in clinical practice [15]. There is concern in the transplant community about the lack of prospective data evaluating the risk of ESKD in donors of African ancestry with a high-risk APOL 1 genotype. A retrospective study found that donors with high-risk APOL1 genotypes had significantly lower pre-donation and post-donation eGFR. However, the rate of eGFR decline was comparable to APOL1-matched non-donor controls [16].

#### Risk of Hypertension

Several adaptive compensatory mechanisms develop post-nephrectomy; kidney plasma volume increases, resulting in glomerular growth and accentuated hyperfiltration. However, hyperfiltration does not cause high glomerular pressure or damage in kidney donors, although albuminuria may occur [17]. A key question is whether nephrectomy affects the prevalence of hypertension. Studies indicate that the prevalence of hypertension increases after kidney donation, with risk varying from zero to a threefold increase. Meta-analyses suggest donor blood pressure rises by 5 mmHg compared to healthy controls [18]. Despite extensive research, varying methodologies challenge the possibility of drawing definite conclusions, as outlined in Table 1.

**TABLE 1:** The complexity in the evaluation of hypertension risk factors.

Blood pressure measurement technique before donation	Home vs. office vs. 24-h measurements. All these methods have been indistinctly applied, without consensus on the most suitable for this population
Hypertension diagnosis before donation	Hypertension does not preclude donation if well-controlled with 2 drugs at the most. The acceptance of a donor candidate is affected by age. The detection of organ damage is a contraindication
Familiar risks	Not recorded in most registries
Smoking	Insufficient data captured in the registries
Comorbidities	Dyslipidemia, abnormal glucose metabolism, or overt diabetes have not been considered in association with hypertension
Overweight	Weight changes after donation have not been systematically reported
Length of follow-up	Most studies report the results from the first 10 years after donation, but long-term data is scarce
Data based on registries	Some registries retrieve data from hospital charts, others from pharmacy repositories, or rely on patients’ reports

Hypertension raises the risk of progressing to ESKD and cardiovascular events; these are reduced if blood pressure is maintained below 130/80 mm Hg after donation [19] because mean blood pressure over 140/80 increases the risk of progressing to ESKD fourfold [12]. The risk of hypertension increases when risk factors such as obesity, smoking, genetic predisposition, older age, and low eGFR accumulate [20]. Weight gain increases the risk of hypertension more in kidney donors than in controls. However, both obese donors and non-donors had a similar hypertension incidence [21].

A BMI >30 doubles the relative risk of ESKD compared to BMI <30 at 15-year follow-up. However, the incidence of ESKD in these groups remain very low (40 cases in BMI >30 vs. 20 cases if BMI <30 out of 10,000 donors) [11].

#### Risk of Death

A recent meta-analysis of over 900 studies found a perioperative mortality rate of 0.01% with low incidence of intraoperative complications (2.3%). With the current laparoscopic nephrectomy technique, the rate of infections, bleeding, and reoperations are quite low. Among these complications, infections are most common and easily treatable [22]. A recent analysis of 164,593 kidney donations reported a death rate 90 days post-surgery less than 1 event per 10,000 donations. Perioperative mortality after living donation declined substantially in the past decade. The risk was higher for male donors and donors with a history of hypertension [23].

Studies in Sweden [24] and the United States [25] have shown that survival is better for live donors compared to the general population. However, when the comparison group consists of healthy individuals from the general population, it is less clear if kidney donation has a detrimental effect on long-term cardiovascular mortality. The previously mentioned U.S. [12] and Norwegian [14] studies present conflicting findings. In Mjøen’s study, the mortality risk ratio was 1.3 for LD compared to controls, and the cardiovascular mortality risk ratio was 1.4. Muzaale reported no increase in long-term mortality risk for donors compared to controls. Both studies face several methodological challenges; for example, in the Norwegian study, the control group consisted of younger individuals, living in rural areas who smoked less and had no family history of kidney disease.

Particular attention should be given to how cardiovascular risk factors change after kidney donation. A small number of studies examined the changes in metabolic factors post-donation [26]. In an Israeli study, LD had higher increases in BMI, triglycerides, type 2 diabetes, and incidence of metabolic syndrome, compared to controls over a five-year follow-up period. Blood pressure was similar between LD and healthy individuals, but paradoxically, cardiovascular events were more common in healthy individuals. A U.S. study also reported similar levels of blood pressure, HbA1c, albuminuria, and lipoproteins in LD and healthy controls after 9 years of follow-up. Noteworthy, LD had higher levels of parathyroid hormone and uric acid, probably because of decreased kidney mass [27].

#### Maternal and Fetal Risks After Kidney Donation

A recent systematic review compiled the results of 16 studies over a 35-year period, including 1,399 post-donation pregnancies [28]. These studies employed different methodologies, and only six of them included a control group. Based on the available evidence, eight clinical practice guidelines, three consensus statements, and four expert-opinion papers were published between 2010 and 2020. The general conclusion is that the occurrence of hypertension during pregnancy increased from 1% to 9% pre-donation or matched controls to 4%–12% post-donation. Pre-eclampsia also increased from 1% to 3% pre-donation or in non-donors to 4%–10% post-donation. The recommendations universally state that women should be counseled about the increased risk of gestational hypertension or pre-eclampsia. Additionally, it should be stressed that, according to the literature, most women had uncomplicated pregnancies post-donation, and the aspiration to have a child should not be seen as a contraindication for donation. In most studies, fetal and neonatal outcomes after kidney donation are like those in non-donor pregnancies.

#### Potential Psychological Consequences of Living Kidney Donation

Live kidney donors may find recovery is hindered by post procedure tiredness, although the majority recover within several months. While 14% of US kidney donors experienced persistent fatigue 1 year after donation, this rate was comparable to healthy controls [29]. Donors with a history of affective disorders, anxiety or lower levels of physical activity were identified as highest risk for persistent fatigue.

Type of surgery does not seem important, with both open and laparoscopic nephrectomy donors experiencing equal mental fatigue and reduced motivation. Although these symptoms had resolved by 3 months, the physical fatigue could persist for up to 12 months [30]. A Dutch study on health-related quality of life (HRQoL), also found that there was no difference in physical scores between pre- and 12 months post-donation [2] but mental scores varied significantly, declining from pre- to 6 months post-donation and then improving from 6 to 12 months. Predictors of greater fatigue included higher baseline fatigue, poorer baseline physical functioning, younger age, longer hospital stays, and greater influence of the recipient’s condition.

Female donors are more affected. A German HRQoL study found similar QoL outcomes across genders, except for the mental component in SF-36, when 51–60-year-old females scored lower than both age-matched males and general female population [31]. This was corroborated by a Norwegian long-term study (217 donors) although fatigue levels were generally low. Here, higher QoL was associated with donors who received recognition whereas donors with regret reported generally elevated fatigue [32]. A Dutch 10-year study reported significant declines in physical function, pain, and general health (SF-36) at follow-up but unfortunately, the lack of a comparator makes it difficult to distinguish the impact of donation from general aging but reinforces the need for psychosocial support [33].

## Long-Term Follow-Up for Kidney Donors

Ever since the first LD kidney transplant in Boston in 1954, the best approach to the care and management of living kidney donors has frequently been debated. As the practice expanded, it was recognized that the health status of kidney donors must be monitored throughout their lives to ensure treatment for preventable illnesses. In current guidelines, yearly living kidney donor follow-up is suggested which includes at the minimum the following: blood pressure, BMI, eGFR, albuminuria, health style, and general wellbeing review [34]. However, a personalized approach is recommended. Compliance with this recommendation may be reduced due to the costs to healthcare organizations. In the US, the Organ Procurement and Transplantation Network requires transplant programs to submit 6-, 12-, and 24-month post-donation follow-up data to the national registry, but after this time point recovering follow-up care costs is billing the recipient’s insurance, while in some cases the programs bill the donor, or the follow-up costs were covered by charitable funds. US researchers advocated for the revision of the Organ Acquisition Cost Center’s policy to include follow-up costs as part of the commitment necessary for living donor care and safety, rather than solely for data collection [35].

Most of the available evidence of LD safety is based on registry data and therefore is only as valid as the reported follow-up, and may be limited, particularly for those who donated more than 20 years ago. Transplant registries are crucial for planning transplant activities, epidemiological analysis, organizing follow-up care, and evaluating outcomes. They are a critical tool for quality control, thus improving patient safety. Altogether, 115 transplant registries are identified worldwide in the International Registry in Organ Donation and Transplantation. Of them, only 16 reported living donor outcomes post-donation including organ function (n = 9) and death (n = 16) [36].

Transplant programs should ensure long-term surveillance of LD, but the dataset captured by different registries is diverse, and its harmonization has proved challenging [37]. Coordinated efforts to gather valuable information from different transplant registries have gained attention recently. In Europe, the European Society of Organ Transplantation launched a platform to host pan-European registries on transplant recipients and living donors. This initiative has the support of the European Commission [38]. Similar efforts are ongoing in the US. [39, 40]. Common barriers to data sharing include technical, economic, legal, and ethical issues [41]. Nevertheless, the efforts outweigh the benefits for patients with kidney disease and donors.

LD often receive care from primary healthcare, the private sector, and occupational health services. Valuable follow-up information emerging from these care providers could enhance the quality of the transplant registries. The digitalization of healthcare provides a unique opportunity for big data analysis, which may improve the understanding of LD clinical outcomes.

### The Evolving Face of Living Kidney Donation

Kidney donation is a safe procedure for carefully screened donors. However, there is uncertainty about the risks of long-term risk when compared to healthy non-donors, especially after the first two decades post-donation. Organ shortage and improved recipient survival after LD transplant are pushing the limits for the acceptability of LD candidates, considering older living donors and those with comorbidities such as impaired glucose tolerance or diabetes without signs of nephropathy. In this context, while careful risk stratification and donor selection remain essential, the inclusion of these potential candidates in the pool represents a promising avenue for expanding living donation. Nevertheless, lifelong LD monitoring to detect treatable problems is paramount. The minimum data proposed to be systematically collected is shown in Table 2. Noteworthy, the targets for these parameters are based on expert opinions. There is no evidence of the impact of managing cardiovascular complications after LD on survival or risk for kidney replacement therapy.

**TABLE 2:** Living donor follow-up checklist.

Variable	Target	To note
Blood pressure	<130/80	The drug of choice is usually on RAAS inhibitors and thiazides. Of crucial importance is a restriction on salt intake under 5g/day
LDL-cholesterol	<2.5 or <1.8 or <1.4	There are no recommendations for kidney donors. The target depends on the underlying conditions and eGFR. Statins are safe
HbA1c	<42 mmol/mol	In case the value is above the normal range, the usual treatment
Creatinine, eGFR	no target	Trends in eGFR should be considered
U-AlbCrea	<3 mg/mmol	A small amount of albuminuria may appear, but if the urine albumin/creatinine ratio is over 60 mg/mmol, a careful evaluation is warranted
Smoking	stop smoking	Usual recommendations
BMI	BMI <25	Usual recommendations
Lifestyle and wellbeing	healthy choices	Usual recommendations
Other important considerations		
Nephrotoxic drugs	Avoidance of NSAIDs. Advice to adjust drug doses to eGFR.	
Complications	Follow-up data and complications, especially severe infections, cardiovascular morbidity, cancer, or psychiatric, should be informed to the local transplant registry administrator	

### Conclusion

This mini-review highlights the uncertainties of LD long-term follow-up, with recommendations and evidence-based targets for managing comorbidities after kidney donation. Further collaborative national and international efforts are needed to advance our knowledge and optimize follow-up care of living kidney donors.
